# Postbiotic production, aggregation properties, binding potential, antioxidants capacity, and functional characterization of the lead *Enterococcus faecium* probiotic strains

**DOI:** 10.3934/microbiol.2025035

**Published:** 2025-11-18

**Authors:** Abrar Hussain, Muhammad Tanweer Khan, Syed Abid Ali

**Affiliations:** 1 Third World Center for Science and Technology, H.E.J. Research Institute of Chemistry, at International Center for Chemical and Biological Sciences (ICCBS), University of Karachi, Karachi, 75270, Pakistan; 2 Wallenberg Laboratory, Sahlgrenska University Hospital, Bruna Stråket 16, SE-413 45, Gothenburg, Sweden

**Keywords:** *E. faecium*, probiotics, postbiotic, *Enterococcus*, biogenic amine

## Abstract

The emergence and applications of probiotic species across industries are growing rapidly, requiring the isolation, identification, and robust characterization of new strains. *Enterococcus faecium*, a dominant species of the genus *Enterococcus*, is widely distributed and has a prominent role in biotechnological applications. The probiotic potential of *E. faecium* is well established, and various strains have been commercially available. In this study, we aimed to provide a strategic road map to explore the probiotic potential, postbiotic production, antioxidant activities, aggregation properties, and functional characterization of the selected *E. faecium* strains (n = 6) isolated locally. All selected strains demonstrated significant probiotic potential, with stress tolerance, aggregation, and postbiotic production. They were free from biogenic amines while exhibiting notable free radical scavenging and reducing activities. Additionally, their ability to adhere to fibrinogen and mucin indicates enhanced potential for mucosal colonization, competitive exclusion of pathogens, and improved host interaction. All strains tolerated digestive stress, two strains (*E. faecium* Se142 and *E. faecium* F25) produced slime, and all exhibited antioxidant activity. The influence of digestive enzymes on enterocins, the production of arginine hydrolases, and the impact of glycine, arginine, and glucose on their growth performance reflected positive attributes. These attributes indicate their potential as ideal candidates for developing new probiotic formulations, with intended food and biotechnological applications. In the future, genomic and *in vivo* validation studies are warranted.

## 1. Introduction

Probiotics are live microorganisms that, when administered in adequate amounts, confer a health benefit to the host. Probiotics have selection criteria, including safety profiling, tolerance potential, aggregation properties, postbiotic and bacteriocins production, and certain application-specific properties [1],[2]. Due to strain-specific effects and a rigorous selection process, only a limited number of microorganisms qualify as probiotics, with lactic acid bacteria comprising most of these approved species [3]. Their safe nature, promising postbiotic productions, enzymatic profiling, and intrinsic characteristics make them important agents in various industrial applications [4]. Probiotics hold a prominent position in the present-day healthcare, biotechnology, veterinary, and food science industries [5],[6]. However, strict regulatory requirements, misleading health claims, and the inherent properties of certain probiotic strains can limit their use, particularly in applications involving humans and animals. Therefore, a comprehensive evaluation of probiotic strains, including their safety profiling, viability under harsh conditions, ability to adhere to host tissues, and aggregation capacities, as well as their potential applications across various sectors, remains essential for ensuring their effectiveness and suitability for use [1].

Lactic acid bacteria (LAB) are the widely explored bacteria for their probiotic potential, dominated by lactobacilli and *Bifidobacterium* species [7],[8]. The genus *Enterococcus*, considered the third-largest genus in the LAB group, is also explored, and probiotic species have been identified. With over 80 identified enterococcal species, the genus has a ubiquitous distribution and is found in every possible environment of microbial existence [9]. The distinguishing characteristics of enterococcal species include genome plasticity, intrinsic tolerance, aggregation properties, bacteriocin (enterocin) production, and survivability under harsh conditions, i.e., high pH, temperature, salinity, and resistance to desiccation [10]. Biochemically, the enterococcal species have thick cell walls, ovoid or round shapes, purple appearance, have no potential to produce oxidase, urease, and catalase enzymes, grow in the presence and absence of air, and are non-motile microorganisms. Enterococci are used in different industries dominated by food, pharmaceuticals, and poultry sectors [11]–[14].

The probiotic potential of enterococcal species is controversial but well-explored. The enterococci are commonly considered an opportunistic pathogen and hence not included in the GRAS (Generally Recognized As Safe) and QPS (Qualified Presumptions of Safety) status microorganisms [9],[15]. The two culprits behind enterococcal pathogenesis are the presence of dozens of virulence traits and antibiotic resistance. Among the enterococcal species, the two species *E. faecium* and *E. faecalis* are dominant, have 70–80% prevalence, are responsible for major enterococcal complications, and are important at the same time [16]. In general, *E. faecium* is considered more resistant, while *E. faecalis* is more virulent [1]. Likewise, the distribution, prevalence, intrinsic properties, and hence applications of these two species are also varying. For instance, *E. faecium* is widely isolated from the food, environment, and agriculture sectors, while *E. faecalis* is identified from hospital settings, medical instruments, and patients with hospital-acquired infections (HAIs) [17]. Additionally, the *E. faecium* species are widely used as probiotics and in food industries, while the positive attribute of *E. faecalis* is less explored due to its virulent nature [16],[18].

Recent advanced technologies classify and differentiate commensal and nosocomial strains of enterococci, providing a better understanding and anticipated applications of the selected species. For example, *Enterococcus faecium* is divided into three clades; A1 is hospital-associated, A2 is animal-related, and clade B is the community-associated clade [14],[15]. Beukers et al. (2017) suggested that different *E. faecium* strains may have the potential to adapt to their respective environments [19]. *E. faecium* species are used in poultry, dairy, food, pharmaceuticals, and probiotics applications. The short generation period, small genomic size and plasticity, and generally safe nature make it an important agent across various applications [9]. Production of single or multiple enterocins, i.e., EntA, EntB, EntP, EntQ, EntL50A/B, and EntSE-K4 are also contributing majorly to the industrial applications of *E. faecium* [13]. Due to the opportunistic nature and sequestration of antibiotic resistance genes, an extensive investigation is crucial for the probiotic potential of *E. faecium*. Although organizations like the European Food Safety Authority (EFSA), the Advisory Committee on Novel Foods and Processes (ACNFP), and the Food Standards Agency (FSA) enabled the usage of *E. faecium* strains as food supplements and additives while following a critical assessment of safety profiling [15].

Various *Enterococcus*-based probiotics are commercially available without being involved in any negative complications, fostering the way to explore more strains for similar potential. In this regard, *E. faecium* SF68® (NCIMB 10415) and *Enterococcus faecalis* Symbioflor 1 are well-described [7]. In previous studies, we screened and identified six (n = 6) *E. faecium* strains from the locally isolated enterococcal biobank from Karachi, Pakistan. The safety, tolerance, and preliminary probiotic properties have been established [1],[12]. Deciphering the full probiotic spectra of these strains, we aimed to fully explore the *in vitro* safety, tolerance capacity, aggregation potential, enzyme production, effect of different substances, antioxidant potential, and binding assays. Elucidation of these properties will not only aid their probiotic potential but can also enhance their industrial applications.

## 2. Materials and methods

### 2.1. Strains selection

In this study, our previously identified *E. faecium* strains with potential probiotic properties were used for comprehensive *in vitro* analysis. The six (n = 6) lead strains, including *E. faecium* NF (RG Pharmaceutica Pvt. Ltd., Karachi), *E. faecium* TWCST1, *E. faecium* TWCST2, *E. faecium* M30, *E. faecium* Se142, and *E*. *faecium* F25, were selected and investigated for probiotic features and industrial applications.

### 2.2. Preliminary probiotic properties and safety assessments

The selected strains were confirmed to be safe, showing no virulence traits or antibiotic resistance at either the phenotypic or genotypic level [12]. The biotechnological properties such as stress tolerance, enzyme production, shelf life, and other properties were also elucidated as described previously [1]. Additional safety assessments of the lead strains were performed that led to exploring their probiotic potential and applications across sectors.

### 2.3. Functional probiotic properties

The potential of the lead strains to produce various bioactive substances was elucidated phenotypically using established protocols as described below.

#### 2.3.1. Slime production

The slime production assay was performed according to Hasan et al. (2018). A modified MRS agar (supplemented with 50 g/L of sucrose) was spotted with fresh culture and incubated for 24–48 hrs at 37 °C. Sticky and gummy colony formation was considered positive for slime production [20]. The same procedure was followed while using the Congo red agar plates (36 g/L sucrose, 0.8 g/L Congo red in 1 liter of BHI agar), as described Kouidhi et al. (2011) [21].

#### 2.3.2. Exopolysaccharide production

The exopolysaccharide (EPS) production was identified using the modified method [22]. The overnight culture was streaked on modified MRS agar (containing 100 g/L sucrose) and incubated at 37 °C for 24 hrs aerobically. Results were analyzed by measuring the size of the colonies, if the colonies are more than 1.5 mm in diameter, they were considered positive for EPS production.

#### 2.3.3. Congo red plate (CRA) assays and the detection of biofilm formation

The qualitative biofilm formation of the lead strains was determined using Congo red as an indicator [23]. A known amount (0.8 g/L) of Congo red was added to basal media (brain heart infusion and Muller Hinton agar), and the overnight cultures were spotted. The plates were kept at 37 °C for two days. The results were analyzed while observing black and red colonies as positive (slime producers) and negative (non-producers), respectively [21].

#### 2.3.4. Rhodamine dye assay for lipase production

The lipase production *via* Rhodamine B degradation was assayed as described by Hasan et al. (2018). The overnight cultures were spotted on a rhodamine agar plate (rhodamine 0.001%, peptone 5 g/L, yeast extracts 3 g/L, agar-agar 15 g/L, NaCl 4 g/L, pH, 7.0 dissolved in DI), supplemented with 31.25 ml/L of olive oil. The plates were incubated at 37 °C for two days and observed under UV radiation at 350 nm. Orange colors around the fluorescent halos were considered positive [20].

#### 2.3.5. Phenol red assay

The phenol red agar assay was performed as described [24]. To the phenol red agar media (in DI water dissolved peptone 5 g/L, yeast extract 3 g/L, agar 15 g/L, CaCl_2_ 1 g/L, and adjusted to pH 7.4 with 0.1 M NaOH), a 10 ml/L phenol red dye (1 mg/ml) and 10 ml/L substrate olive oil was added. Plates were spotted with the overnight cultures and incubated at 37 °C for 2 days. Change of color from orange to pink indicated positive results.

#### 2.3.6. Fermentation of non-digestive sugars

The fermentation profiling of digestion resistance sugars was performed using the method described previously [25]. A total of 20 g/L raffinose (D-Raffinose pentahydrate, Aldrich Chemical Company, Inc. USA) was added to MRS agar, and the plates were spotted with overnight cultures followed by incubation at 37 °C for 24 hrs. The change in color from red to yellow showed positive results.

#### 2.3.7. β-galactosidase assay

The quantitative analysis of β-galactosidase was elucidated using the altered method of Vasudha et al. (2023) [26]. Overnight, cultures were centrifuged at 10,000 × g for 10 minutes at 25 °C. Pellets were washed and resuspended in PBS (pH 7.0). A 150 µL aliquot was transferred to a sterile 96-well plate, and initial absorbance was recorded at 560 nm (OD_A560_). Cells were permeabilized by adding toluene/acetone (1:9 v/v) and vortexed. For the reaction, 900 µL PBS (pH 7.0) and 200 µL o-Nitrophenylβ-D-galactopyranoside (ONPG, 4 mg/mL) (Baker Organic Chemicals; 2146) were added to an empty tube, followed by 100 µL of the permeabilized cell mixture. The reaction mixture was incubated at 37 °C for 1 hour, then terminated with 1 M Na_2_CO_3_. The final absorbance was measured (OD_B_ 560 nm), and the percent β-galactosidase production was determined as follows (Eq 1).

$$\%\text{ β}-\text{galactosidase production}=\left(\frac{A-B}{A}\right)\times 100$$ (1)

where A is the absorbance before the incubation and B is the absorbance after incubation.

### 2.4. Safety profiling

#### 2.4.1. Biofilm formation on polystyrene

The biofilm formation potential of the lead strains was evaluated by a method described earlier [27]. The overnight culture grown in TSB (Tryptone Soya Broth, CM0129, Oxoid) supplemented with 0.5% glucose was added (1:40 diluted) to a sterile 96-well polystyrene plate and incubated for 24 hrs at 37 °C. After incubation, the plate was washed thrice with phosphate buffer saline (PBS, pH 7.0) and removed the planktonic cells. The plate was dried on tissue beds for 15 min, followed by staining with 0.1% (w/v) safranin for 1 min. The dye was washed with deionized water, and OD_490_ was recorded using a spectrophotometer (Multiskan GO, Thermo-Scientific, Finland). The uninoculated broth and *Enterococcus faecalis* ATCC29212 were used as negative and positive controls respectively. The results were presented as non-biofilm (OD below 0.081), moderate biofilm (≥ 0.081), and high biofilm (≥ 0.2) former.

#### 2.4.2. Biogenic amines detection

The production of biogenic amine was elucidated using the modified method as described earlier [28]. A total of 0.2% solution of different amino acids, cysteine, isoleucine, arginine, tryptophan, threonine, histidine (Merck), and ornithine and citrulline (Sigma) was made in DI water. Two µL of the amino acid solution were added to 1 mL of the decarboxylase media, and 100 µL/well of this mixture was added to a sterile 96-well plate and supplemented with 50 µL of the overnight culture. The plate was incubated aerobically at 37 °C for two days, and the biogenic amine production was inspected visually via the color change from yellow to purple. Overnight culture in decarboxylase media without amino acids was used as a control. The experiment was repeated three times.

### 2.5. Antioxidant and reducing potential determination

The antioxidants and reducing potential of the selected strains were determined while elucidating their DPPH, OH^-^ radical, and reducing potential.

#### 2.5.1. Antioxidant activity (DPPH scavenging potential)

The antioxidant potential of the selected enterococcal strains was determined using DPPH as a substrate, as reported by Kim et al. [29] with little modification. The overnight culture was harvested, and the pellets were suspended in PBS (pH 7.0). A freshly prepared 200 µL methanolic solution of 0.1 mM 2,2-diphenyl-1-picrylhydrazyl (DPPH) was added to 800 µL of the selected strain and incubated in the dark for 30 min at 37 °C. After incubation, the solution was centrifuged and measured the absorbance at 517 nm. One mg/mL of ascorbic acid and methanol was used as a positive and negative control, respectively. The DPPH antioxidant activity was measured as (Eq 2)

$$\text{\% Scavenging activity}=1-\frac{As}{Ac}\times 100$$ (2)

where, As and Ac are the absorbance of sample and control, respectively.

#### 2.5.2. Hydroxyl (OH^-^) radical scavenging ability

The hydroxyl radical scavenging potential was measured as described [30], with slight modification. The cell-free supernatant (CFS) was obtained by harvesting the overnight MRS-grown culture at 10,000 g for 10 min at room temperatures, and the supernatant was then filtered using 0.22 µm filters. To obtain the cell extracts (CEs), the pellets were washed two times with sterile PBS (pH 7.4) and resuspended in it, followed by cell disruption with ultrasonication (Vibra-Cell™) in water (6 mm probe, 5 s on/off cycles, 300 W, for 5 min). The mixture was centrifuged at 8000 g for 10 min, and the resulting supernatant was collected as cell-free extracts. Likewise, the overnight BHI-grown culture was directly used as intact cells (IC).

For hydroxyl scavenging potential, 0.5 mL of the overnight culture (intact cells), 0.5 mL of cell-free-supernatant (CFS), and 0.5 mL of cell extracts (CEs) were mixed with the mixture of ferrous sulfate, salicylic acid, and hydrogen peroxide (each with 9 mM and 0.5 mL) in a separate vial and thoroughly mixed. The mixtures were then incubated at 37 °C for 30 min, and their absorbance (OD = 510 nm) was measured. A known amount (0.5 mL) of the above solution without culture was used as a control. The percentage of hydroxyl radical scavenging activity was calculated as above.

#### 2.5.3. Reducing potential

The reducing potential was determined following the reported method of Wu et al. [30]. Briefly, 100 µL of the ICs, CFS, and CEs (prepared as above) were mixed with 100 µL of 1% (w/v) potassium hexacyanoferrate (III) and 100 µL of 0.2 M phosphate-buffered saline (PBS, pH 6.6) as a separate independent experiment. All the mixtures were incubated in a water bath (50 °C) for 20 min and cooled at room temperature. After cooling, 100 µL of a 10% (w/v) trichloroacetic acid was added and centrifuged at 8000 g for 7 min. The obtained supernatant was mixed with 100 µL of 0.1% (w/v) FeCl_3_ solution and 100 µL of sterile water (as a control). The mixture was incubated at room temperature for 10 min and absorbance at 700 nm was measured. The reducing activity was calculated as follows (Eq 3).

$$\text{Reducing activity}={\text{A}}_{1}-{\text{A}}_{0}$$ (3)

where A_0_, absorbance of sterile water, and A_1_ is the absorbance of the sample (FeCl_3_).

#### 2.5.4. ABTS radical scavenging potential

The potential of 2,2-Azino-Bis (3-Ethylbenzothiazoline)-6-Sulfonic Acid (ABTS) radical scavenging potential of the selected strains was determined as described [30]. A 0.007 M/L solution of ABTS and a 0.0024 M/L solution of potassium persulfate (K_2_S_2_O_8_; Merck) were prepared in DI water. Equal volumes of both solutions were mixed and incubated in the dark at room temperature for 12–15 hrs. From this stock solution, 200 µL was taken and diluted with DI water until absorbance of 0.70 ± 0.05 at 734 nm. Overnight cultures of the selected strains were centrifuged at 10,000 g for 5 min and washed with PBS and resuspended in it. This suspension was mixed (1:2 ratio) with the above mixture and incubated for 30 min in the dark at RT, followed by measuring absorbance at 734 nm. PBS was used as a control. The percent ABTS scavenging potential (Eq 4)was measured as follows:

$$\%\;ABTS\;scavenging\;potential=(1-\frac{A1}{A0})\times 100$$ (4)

where A_1_ is the absorbance of the sample and A_0_ is the absorbance of the control.

### 2.6. Aggregation, adhesion, and binding assays

Adhesion to host epithelial cells is an important trait in the selection of robust probiotic strains and is widely recognized as a fundamental criterion for strain selection. This ability supports oral or gut adherence/colonization, which is crucial for mediating health-promoting effects [31]. In continuation of the earlier work, we explored additional characteristics to further validate the probiotic attributes of the lead strains.

#### 2.6.1. Visual auto-aggregation

The visual auto-aggregation was determined by the published method in [32], with slight modification. The overnight culture of both selected (probiotics) and pathogenic strains was grown and centrifuged at 6000 g for 15 min. The pellets were washed twice with PBS (pH 7.4) and resuspended in it. The initial OD_600_ was set as 0.5 ± 0.05, and equal volumes (200 µL) of both strains were mixed, vortexed, and incubated at 37 °C for 24 hrs. The degree of aggregation was recorded on an arbitrary scale of 0 (no visible aggregates), +1 (smaller aggregation), +2 (aggregates that are seen but do not settle), +3 (large aggregates that settle and show low turbidity in the suspension), and +4 (large aggregates that settle immediately and show no turbidity).

#### 2.6.2. Auto-aggregation *via* Dynamic Light Scattering (DLS) analysis

The dynamic light scattering (DLS) analysis was performed as described in [33] with modifications. The optical density (OD) of *E. faecium* TWCST1 overnight culture was adjusted to 0.2 at 600 nm using a spectrophotometer. Particle size and polydispersity index (PDI) was analyzed using a Laser Spectroscatter-201 system (RiNA GmbH, Berlin, Germany) equipped with a He-Ne laser (1050 mW output, 690 nm wavelength). A 15 µL aliquot of the culture was placed in the measurement cell and fifty measurements were taken at room temperature with a 1-second await and a 20-second auto-piloted run. Light scattering was recorded at a fixed angle of 90°. The CONTIN algorithm was used to determine the hydrodynamic radius and autocorrelation function [34]. Readings were taken at 0, 1, and 24 hrs. Data analysis was performed using PMgrv3.01 software. The degree of auto-aggregation was shown by an increase in the polydispersity index.

#### 2.6.3. Aggregation substances detection

The production of enterococcal aggregation substances (AS) was determined by clumping assay as described by Bhardwaj et al. (2011) with slight modification [35]. Test and pathogenic strains were grown in Todd-Hewitt Broth (THB) for 24 hrs, followed by centrifugation (10,000 g for 15 min) and sterilization by 0.22 µm filters. Equal volume of test strain's supernatants, pathogenic strains, and THB were taken and incubated at 37 °C for 24 hrs. The results were examined visually at two-hour intervals. The assay was also performed using a spectrophotometric method, and the OD was measured at 600 nm at 2 hr intervals.

#### 2.6.4. Fibrinogen binding assay

The fibrinogen adhesion potential of the selected strains was elucidated following the method of Collins et al. (2012) with minor modification [36]. Bovine plasma fibrinogen (10 mg/mL) was dissolved in 50 mM sodium carbonate buffer (pH 9.6). 100 µl of this solution was added to a sterile 96-well plate and incubated overnight at 4 °C. After incubation, the unbound fibrinogen was removed by washing with PBS three times, followed by the blockage of wells with the addition of 100 µL of 5% (w/v) bovine serum albumin solution to the treated and untreated wells. Unbound BSA was removed by washing with PBS. The overnight bacterial culture was harvested (10,000 g for 5 min) and washed once with PBS (pH 5.0) and measured the initial absorbance. A total of 100 µL of these suspended bacterial cells were added to the wells (both coated with fibrinogen and BSA and BSA only) and incubated for 2 hrs at 37 °C. After incubation, the wells were washed three times with PBS (pH 5.0), followed by the fixing of adherent cells with the addition of 100 µL of 25% formaldehyde while being incubated at room temperature for 20 min. After fixation, formaldehyde was removed by washing the wells three times with PBS. The adherent bacteria were stained with 1% crystal violet for 1 minute at room temperature. Wells were then thoroughly washed again with PBS to remove excess stain. The retained crystal violet was solubilized in 100 µL of 5% (v/v) acetic acid. Absorbance was measured at 570 nm (OD_750_) using a microplate reader. Bacterial adhesion (Eq 5)was calculated in relative absorbance units as follows:

$$\text{Abundance }\left(\text{OD}570\right)=\frac{\left(A-B\right)}{A}\times 100$$ (5)

where A is the absorbance of fibrinogen + BSA + bacteria, and B is the absorbance of BSA + bacteria.

#### 2.6.5. Mucin adhesion potential

The mucin adhesion potential of the selected strains was determined using the described protocol [37] with slight modification. Briefly, 100 µl of mucin solution (10 mg/mL in PBS) was added to a sterile 96-well plate and incubated overnight at 4 °C. On the next day, the wells were washed twice with PBS (pH 7.0), and 100 µL of 20 mg/mL (w/v) of bovine serum albumin was added, followed by 4 hrs incubation at 4 °C. Again, it was washed with PBS, and 100 µl of an overnight culture was added. The plate was incubated at 37 °C for 1–2 hrs and washed three times with PBS, followed by treatment with 100 µL of 0.5% Triton X-100 for 2 min. The mixture was removed, and the plate was kept at room temperature in an orbital shaker (TRM-4 thermo shaker) at 150 rpm for 10 min. After releasing the adhered cells from the wells, 3 µL was streaked on MRS agar and incubated at 37 °C for 48 hrs aerobically. The results were reported as cfu/mL.

### 2.7. Stress tolerance

Stress tolerance potential is one of the key criteria in probiotic strain selection. In this study, we aimed to evaluate the tolerance potential of selected strains against various stress-inducing substances, using a range of analytical methods as described below.

#### 2.7.1. Trypsin tolerance

Tolerance potential against trypsin (MP Biomedical, Inc., lot no. 1654J) was evaluated as described previously [38]. Different concentrations, i.e., 0.5%, 1.0%, 1.5%, and 2.0% (w/v) of trypsin, were prepared in DI water. Approximately 2% of the overnight cultures were added to the sterile 96-well plate and measured their absorbance for 4 hrs at 600 nm, with 1 hr intervals, while incubated at 37 °C. Culture without the addition of trypsin was used as a positive control. Growth measurement was calculated as OD versus time and percent reduction in the growth was determined (Eq 6)

% Reduction=(ODc−ODt)×100 (6)

where OD_c_ and OD_t_ refer to the OD of the control and treated samples, respectively. Reduction was calculated using culture and treated sample after 4 hrs of incubation.

#### 2.7.2. Simulated pancreatic juice (SPJ) tolerance

The SPJ was prepared by dissolving bile (3 g/L), pancreatin (0.1 g/L), sodium phosphate dibasic heptahydrate (50.81 g/L), and NaCl (8.5 g/L) in a KH_2_PO_4_ buffer of pH 8.0. The assay was performed as described [26] with modifications. Overnight cultures were harvested by centrifugation at 6000 × g for 20 minutes at room temperature, washed with PBS, and resuspended in SPJ. The suspension was incubated at 37 °C for 3 hrs. After incubation, aliquots were streaked on MRS agar and incubated at 37 °C for 48 hrs. The percent survival rate was calculated based on colony-forming units (CFUs) before and after SPJ exposure as follows (Eq 7):

$$\%\;survival\;rate\frac{\frac{cfu}{ml}in\;SPJ}{\frac{cfu}{ml}in\;control}\times 100$$ (7)

Overnight culture without SPJ treatment was used as a control.

#### 2.7.3. Ethanol tolerance

The ethanol tolerance of the selected strains was determined as described earlier [39] with modification. The selected strains were grown in BHI broth and added to the different concentrations (v/v%) of ethanol i.e., 0%, 5%, 10%, and 15%. The suspension was added to the sterile 96-well plate, and absorbance (OD_600_) was measured at 1 hr intervals for 4 hrs. The growth curve was determined.

#### 2.7.4. Tolerance to hyaluronic acid

Tolerance against hyaluronic acid was evaluated as described by [40], with little modification. The overnight culture was grown in BHI broth and kept at 37 °C for 24 hrs, followed by centrifugation (10,000 g for 10 min) and washing with PBS (pH 7.0). Different concentrations of hyaluronic acids i.e., 0.1, 0.5, 1, and 2 mg/mL, were prepared and dissolved the pellets. A total of 150 µL of the suspension was added to 96-well plate, and the growth pattern was measured at 600 nm for 4 hrs.

### 2.8. Functional profiling

To elucidate and identify possible food, biotechnological, and other industrial applications, functional properties were elucidated.

#### 2.8.1. Effect of digestive enzymes on enterocin production

The effect of digestive enzymes on the potential of enterocins was carried out using a modified method [41],[44]. Cell-free supernatants (CFS) were prepared by harvesting (10,000 g for 5 min) the fresh culture and keeping it at 80 °C for 10 min to remove the leftover cells. Different enzymes, i.e., pepsin (0.1 mg/mL) and proteinase-K (100 µL/mL), were added to the CFS and incubated for 60 min at 37 °C, followed by heating at 98 °C for 5 min. The fresh test strains (*Proteus mirabilis*, *Enterobacter cloacae* ATCC 13047, *L. monocytogenes* ATCC 13932, *Pseudomonas aeruginosa* ATCC 27853, *Micrococcus luteus* ATCC 10240, *Staphylococcus aureus* ATCC 25923, *E. faecium* ATCC 6569, and *Enterococcus faecalis* ATCC 51299) were added to a 96-well plate, and their absorbance was measured at 600 nm with three-hour intervals. After 6 hr of incubation, the modified CFS was added to the test strain and followed for 24 hrs.

#### 2.8.2. Screening for arginine hydrolase enzymes

The presence of arginine hydrolase enzymes in the selected strains was carried out using the methods of Khushboo et al. (2023) with slight modification [43]. The arginine hydrolase broth was prepared as follows: L-arginine 10 g/L, NaCl 5 g/L, agar 3 g/L, peptone 1 g/L, K_2_HPO_4_ 0.30 g/L, bromo cresol purple 0.016 g/L, and pH was adjusted to 6.0, and sterilized by autoclaving at 121 °C for 15 min. For the experiment, 100 µl of overnight culture were transferred to 1 mL of the media and incubated at 37 °C for 24 hrs. The initial change in color from purple to yellow indicated the consumption of glucose. The suspension was kept again for another 24 hrs (to give sufficient time for bacteria to utilize the arginine), and observed color changes from yellow to purple, indicating the production of arginine hydrolase enzymes. The experiment was performed three times, and media without culture was used as a negative control.

#### 2.8.3. Impact of arginine, glucose, and glycine on the growth

The growth performance of the selected strains in the presence of arginine, glucose, and glycine (25 mM concentration each) was determined as described by Snell et al. (2024) with slight modifications [42]. The BHI-grown overnight cultures of the selected strains were diluted 1:10 in a 25 mM solution of arginine, glucose, and glycine. A total of 150 µl of this suspension was added to a sterile 96-well plate, and their growth curve was plotted at a 3 hr interval for 24 hrs, incubated at 37 °C aerobically, while absorbance was measured at OD_600_. Overnight culture without treatment solution was used as a control.

To measure the colony forming unit per mL (cfu/mL) of the selected strains in the presence of these solutions, 100 µL of the overnight culture was added to 900 µL of the solution and incubated at 37 °C for 24 hrs. After incubation, the treated cultures were grown on MRS agar and again incubated for 48 hrs aerobically. The cfu/mL was counted considering the overnight culture as log10^7^ cfu/mL.

#### 2.8.4. Lactose and glucose consumption (KIA test)

To determine the glucose and lactose consumption, the selected strains were subjected to the KIA test following the protocol by Khushboo et al. (2023) with little modification [43]. Kliger's Iron Agar (KIA) (Lab-Lemco powder (meat extracts) 3 g/L, yeast extract 3 g/L, peptone 20 g/L, sodium chloride 5 g/L, lactose 10 g/L, glucose 1 g/L, ferric citrate 0.3 g/L, sodium thiosulphate 0.3 g/L, phenol red 0.05 g/L, agar 15 g/L, pH 7.4 ± 2) was prepared and sterilized by autoclaving. The slants were prepared in 10 mL slants tubes and kept for solidification. An overnight culture was inoculated while streaking the slant and stabbing the butt. The slant tubes were incubated at 37 °C for 24 hrs. The results were analyzed by changing the color of the slants and butt and noted as follows: If the slant was yellow (acidic) and the butt was red (alkaline), it indicated that the glucose was fermented; if both the slant and butt were yellow (acidic), it reflected that both glucose and lactose were fermented; if both the slant and butt were alkaline (red), it indicated that neither glucose or lactose were fermented; if the media turned black, it indicated the production of hydrogen sulfide (H_2_S) gas; and if the media in the slants became cracked, gaps occurred, or bubbles were produced, it indicated the production of gas.

### 2.9. Statistical analysis

In this study, each experiment was independently performed three times, and the results are expressed as mean ± standard deviation (SD). Due to our focus on the reproducibility and consistency of findings across independent experiments and exploring their probiotics potential, we expressed data as the mean of three experiments.

## 3. Results

### 3.1. Strain selection and preliminary probiotic properties

The selected strains were grown in freshly prepared BHI or MRS media and used in further experimentations.

### 3.2. Production potential

The experimentally determined characteristics and their implications for enterococcal probiotics are described.

#### 3.2.1. Slime production

The lipase production was analyzed by slime production capacity. After two days of incubation, *E. faecium* Se142 and *E. faecium* F25 were identified positive, while all others showed negative results (Table 1). Slime production via the Congo red assay reflected black colonies for *E. faecium* Se142, indicating its slime production, while all other strains were declared negative (Figures S1A-B).

**Table 1.** Phenotypic characteristics of selected *E. faecium* strains based on various probiotic-related *in vitro* assays.

		Selected strains		

Properties		*E. faecium* NF	*E. faecium* TWCST1	*E. faecium* TWCST2	*E. faecium* M30	*E. faecium* Se142	*E. faecium* F25
Slime production		-	-	-	-	+	+
EPS productions		+	-	++	+	+	+
Biofilm via CRA		-	-	-	-	-	-
Rhodamine degradation		-	-	-	-	-	-
Phenol red assay		-	-	-	-	-	-
Fermentation of raffinose		+	+	+	+	+	+
▪Mucin binding potential log_10_^7^ cfu/mL		55.33	67	73	66.33	68.33	59
*Growth performance log_10_^7^ cfu/mL	Arginine	57.5	46	45	44	48.5	35
	Glucose	64	63	71	66.5	40	27
	Glycine	53	56	59.5	47	65	79.5

+; producers, -; non-producers, CRA; Congo red assay, *growth performance was checked against arginine, glycine, and glucose (25mM each). ▪log_10_^7^ cfu/mL, considering the value of an overnight culture.

#### 3.2.2. Exopolysaccharide (EPS) production

The selected strains had the potential to produce EPS. All strains, except *E. faecium* TWCST1, were positive for EPS production (Table 1). *E. faecium* M30 showed moderate production, while *E. faecium* TWCST2 showed greater production, as shown in Figure S1C. *E. faecalis* ATCC 29212 and *Enterobacter cloacae* ATCC 13947 were used as positive controls.

#### 3.2.3. CRA and the detection of biofilm formation

The biofilm formation potential of the selected strain was observed using Congo red as a substrate and Muller Hinton Agar as basal media. After two days of incubation, only red colonies were observed, indicating the non-biofilm forming status of our strains (Table 1; Figure S1D).

#### 3.2.4. Rhodamine dye degradation assay

The analysis of rhodamine dye degradation (lipase production) was analyzed under UV after two days of incubation. The results indicated that all selected strains were unable to produce an orange, fluorescent zone around the colonies, thus considered negative (Figure S1E).

#### 3.2.5. Phenol red dye assay

The phenol red assay using olive oil as a substrate was assessed after two days of incubation. The results indicated the absence of pink color in the selected strains, reflecting the negative results of the test (Figure S1F).

#### 3.2.6. Fermentation of non-human digestive sugars

The fermentation of raffinose was observed after 24 hrs of incubation at static conditions. The plate result showed a positive result for all selected strains (Figure S1G).

### 3.3. Safety assessment

In addition to the established safety profiling of these strains, further tests were conducted to fully explore their probiotic potential and industrial applications.

#### 3.3.1. Biofilm formation on polystyrene

The qualitative and quantitative biofilm formation of the selected strains was analyzed using a polystyrene microtiter plate. The obtained OD was in the range of non-biofilm formers, i.e., below 0.081, reflecting the strains safety (Figure S1H).

#### 3.3.2. Biogenic amines production

The results of biogenic amines were elucidated after two days of aerobic incubation. The results showed that all strains were unable to produce a pinkish color, and thus unable to produce the biogenic amines (Figure S2A).

### 3.4. Determination of antioxidants and reducing potential

#### 3.4.1. DPPH scavenging potential

**Figure 1.**
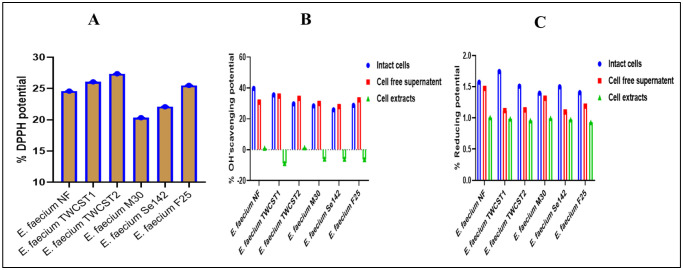
(A) Percent antioxidant activity of the selected *E. faecium* strains against 0.1 mM DPPH, indicating free radical scavenging capacity. (B) Hydroxyl radical scavenging activity (%) of the selected strains assessed using whole culture, cell-free supernatant, and cell extract preparations. (C) Ferric reducing antioxidant potential assay results, indicating low ferric ion reducing capacity of the selected strains.

The antioxidant potential of the selected enterococcal strains against 2,2-diphenyl-1-picrylhydrazyl (DPPH) reflected promising scavenging potential. All selected strains exhibited comparable antioxidant activity, ranging from 20% to 27% (Figure 1A). The lowest activity was shown by *E. faecium* M30 (20.34%), while the highest was shown by *E. faecium* TWCST2 (27.37%).

#### 3.4.2. Hydroxyl (OH^-^) radical scavenging ability

The hydroxyl (OH^-^) radical scavenging potential of the selected *E. faecium* strains were evaluated using three preparations: Intact cells (overnight culture), cell-free supernatants, and cell extracts. The highest scavenging potential was identified in intact cells, with *E. faecium* NF showing 39.8% activity, followed by *E. faecium* TWCST1 at 35.5%. The lowest activity in this group was shown by *E. faecium* Se142 (25.8%). Cell-free supernatants demonstrated relatively consistent scavenging activity across strains, ranging from 29.9% (*E. faecium* M30) to 34.7% (*E. faecium* TWCST1). In contrast, cell extracts exhibited minimal or no scavenging potential. Only *E. faecium* NF (1.05%) and *E. faecium* TWCST2 (1.36%) showed measurable activity, while the remaining strains displayed negligible or negative values (Figure 1B).

#### 3.4.3. Reducing capacity

The ferric reducing antioxidant power (FRAP) of the selected *E. faecium* strains exhibited generally low reducing potential across all tested preparations, i.e., intact cells, cell-free supernatants, and cell-free extracts (Figure 1C). The reducing activity for ICs and CFS ranged from 1.0% to 1.5%, while CFEs showed slightly lower values, ranging from 0.92% to 1.0%. Among all strains, *E. faecium* NF demonstrated the highest reducing potential across all three sample types.

#### 3.4.4. ABTS radical scavenging potential

The oxidation of ABTS with potassium persulfate was producing ABTS^+^, which, upon reaction with an H^+^-donating agent, became decolorized (from blue to colorless). This potential was determined and expressed as percent scavenging activity. The result analysis reflected good ABTS scavenging potential of the selected strains (Figure 4C). The percent scavenging potential almost remain in the same range (62.77, 62.82, and 62.29%) for *E. faecium* M30, Se142, and F25 respectively, while the other three strains showed variation, i.e., *E. faecium* TWCST1 (56.46%), *E*. faecium TWCST2 (40.08%), and *E. faecium* NF (49.10%).

### 3.5. Adhesion and binding potential

#### 3.5.1. Visual auto-aggregation and Dynamic Light Scattering analysis

The auto-aggregation potential of the lead *E. faecium* strains was assessed visually after 24 hrs of incubation, with aggregation levels assigned arbitrary scores ranging from +1 (no aggregation) to +4 (highest aggregation). All strains exhibited a score of +3, indicating moderate aggregation with low turbidity, whereas *E. faecium* NF showed a score of +4, reflecting higher turbidity and stronger aggregation (Figure S2B).

**Figure 2.**
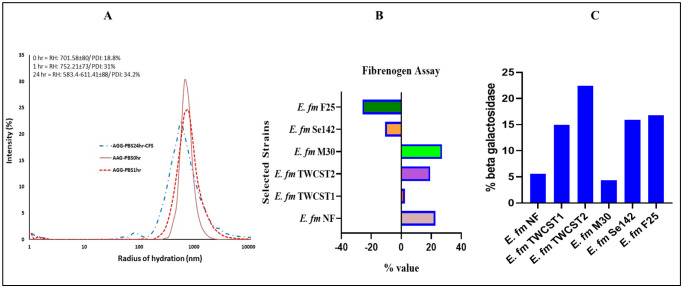
(A) Dynamic light scattering (DLS) analysis of *E. faecium* TWCST1, showing an increase in polydispersity index (PDI) after 24 hrs of incubation, indicating greater heterogeneity in particle size distribution. (B) Fibrinogen-binding potential of the selected *E. faecium* strains, showing both positive and negative binding potentials. (C) β‑Galactosidase activity in the selected strains, expressed as percent production, with moderate levels observed using O‑nitrophenyl‑β‑D‑galactopyranoside (ONPG) as a substrate.

To further validate the visual observations, Dynamic Light Scattering (DLS) analysis was conducted using *E. faecium* TWCST1 as a representative strain. The DLS results revealed an increase in hydrodynamic particle size over time, indicative of auto-aggregation behavior. The polydispersity index (PDI) increased from 18.8% at 0 hr to 31% after 1 hr of incubation, with no significant change thereafter (34.2% at 24 hrs). Additionally, a bathochromic shift accompanied by spectral broadening was observed after 24 hrs, further supporting the strain's aggregation potential (Figure 2A).

#### 3.5.2. Aggregation substances (AS) detection

The production of aggregation substances and co-aggregation potential of the selected strains were assessed visually and spectrophotometrically. Our results indicated that the test strains did not exhibit co-aggregation with pathogenic strains. In contrast, the control strains *E. faecalis* ATCC 51299 and ATCC 29212 demonstrated aggregation with *Pseudomonas aeruginosa*, as shown in Figure S3A. Spectrophotometric analysis revealed no visible clump formation in the test strains up to 6 hrs of incubation. After 24 hrs, slight clumping was observed in *E. faecium* M30 and *E. faecium* TWCST1 when co-incubated with *E. faecalis* ATCC 51299. A gradual increase in aggregation with *P. aeruginosa* and *Staphylococcus aureus* was also observed in the presence of *E. faecalis* ATCC 51299. Similar aggregation patterns were noted when *E. faecalis* ATCC 29212 was used as the counter strain (Figure S3B).

#### 3.5.3. Fibrinogen binding assay

The fibrinogen binding capacity of the selected strains was evaluated, showing both positive and negative binding potentials (Figure 2B). The highest binding was observed in *E. faecium* M30 (27.21%), followed by *E. faecium* NF (22.75%). In contrast, *E. faecium* Se142 and *E. faecium* F25 exhibited negative binding values, measuring -10.75% and -25.89%, respectively.

#### 3.5.4. Mucin adhesion capacity

To assess mucin adhesion, the mucin-binding potential of the selected strains were evaluated by quantifying viable bacterial counts (cfu/mL). The experiment was performed in triplicate, and the mean values were normalized to the cfu/mL of overnight cultures. The results demonstrated that all strains exhibited strong mucin-binding capacity, with relatively close adhesion levels ranging from 55 to 73 log_10_^7^ cfu/mL (Figure S4). The highest mucin-binding potential was observed in *E*. *faecium* TWCST1, followed by *E. faecium* Se142, whereas *E. faecium* NF and *E. faecium* F25 showed comparatively lower adhesion. The detailed cfu/mL values for all strains are summarized in Table 1.

#### 3.5.5. Percent production of β-galactosidase

The β-galactosidase production capacity of the selected strains was quantified spectrophotometrically after 1 hour of incubation. All strains demonstrated moderate enzyme production, ranging from 5.5% to 22.4% (Figure 2C). The highest β-galactosidase activity was detected in *E. faecium* TWCST2 (22.47%), followed by *E. faecium* F25 (16.7%). In comparison, the commercially available control strain exhibited significantly lower activity, with only 5.58% enzyme production.

### 3.6. Stress tolerance

Tolerance potential against different substances were carried out, and the results were analyzed. The ranges, values, and duration, etc. were selected as documented in the literature.

#### 3.6.1. Trypsin tolerance

**Figure 3.**
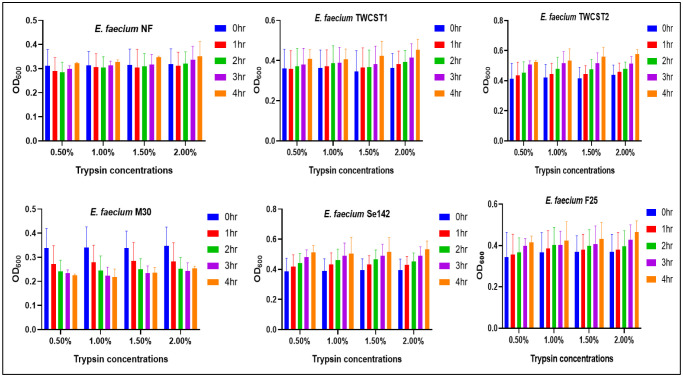
Tolerance of the selected *E. faecium* strains to varying concentrations of trypsin, evaluated by measuring survival or growth after exposure.

**Figure 4.**
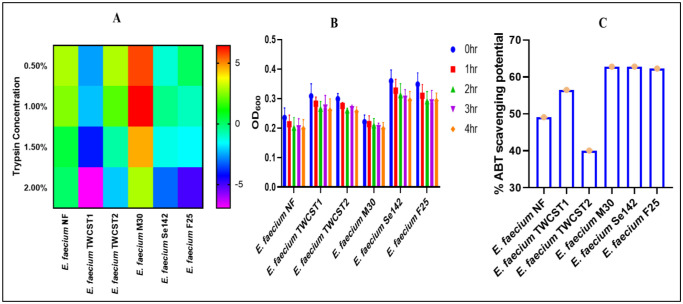
(A) Heatmap illustrating trypsin tolerance of the selected *E. faecium* strains, showing percent reduction in growth following 4 hrs of exposure to varying trypsin concentrations. (B) Tolerance of the selected strains to simulated pancreatic juice after 4 hrs of incubation, indicating survival potential under gastrointestinal conditions. (C) The percent scavenging potential of the selected strains using 7mM ABTS.

Tolerance against varying concentrations of trypsin was assessed by incubating the strains at 37 °C for 4 hrs. The results demonstrated strong trypsin tolerance across all tested strains. No significant impact on bacterial growth was observed with increasing trypsin concentrations in most strains, except for *E. faecium* M30, which exhibited a gradual decline in growth correlating with increasing trypsin levels (Figure 3). The percentage reduction in growth after 4 hrs of incubation was also evaluated. Heat map analysis (Figure 4A) revealed the highest reduction (6.45%) at 1.0% trypsin concentration in *E. faecium* M30. In contrast, no significant growth reduction was noted in other strains, except *E. faecium* NF, which showed a moderate decrease ranging from 0.02% to 2.8%.

#### 3.6.2. Simulated pancreatic juice (SPJ) tolerance

The selected strains were treated against SPJ for 4 hrs and measured their absorbance at one-hour interval. The results indicated strong tolerance potential, as only a slight, gradual reduction in growth was observed with increasing incubation time (Figure 4B).

#### 3.6.3. Ethanol stress

**Figure 5.**
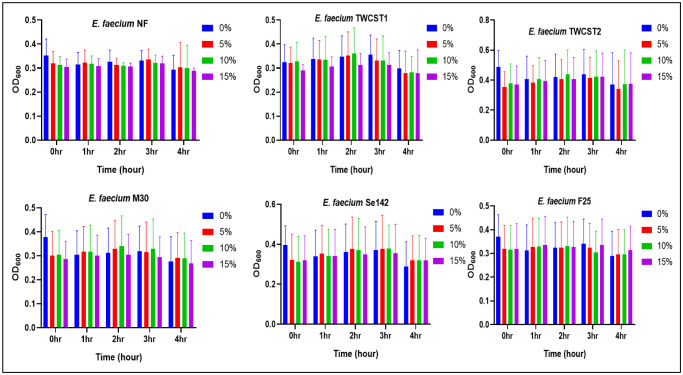
Tolerance of the selected *E. faecium* strains to varying ethanol concentrations, showing minimal reduction in absorbance after 4 hrs of exposure to 15% ethanol.

The tolerance potential of the chosen strain against various concentrations of ethanol revealed relatively consistent survivability. A slight, gradual decline in growth was observed at 15% ethanol, becoming more pronounced after 4 hrs of incubation. Notably, *E. faecium* TWCST1 and *E. faecium* M30 showed the most significant reduction in absorbance at the 4 hr time (Figure 5).

#### 3.6.4. Hyaluronic acid tolerance

**Figure 6.**
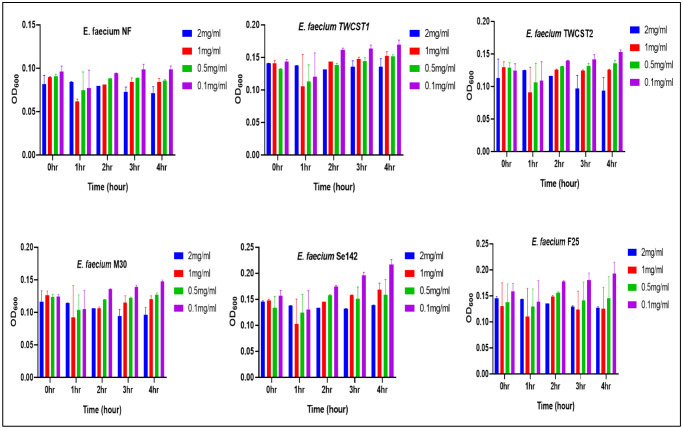
Tolerance of the selected *E. faecium* strains to various concentrations of hyaluronic acid, indicating a lower reduction in the strains survivability when the concentration is rising.

Tolerance against different concentrations of hyaluronic acid (HA) was determined after 4 hrs with one-hour intervals. The results showed that all selected strains were able to survive across the tested HA concentrations (Figure 6). Maximum growth was recorded at the lowest concentration (0.1 mg/mL), while higher HA concentrations were associated with reduced bacterial growth. Interestingly, incubation time had no significant effect on the overall survivability, as similar growth patterns were observed at 0 and 4 hrs. In all strains, a slight reduction in growth was noted at the 1 hour, after which the levels remained relatively stable.

### 3.7. Functional profiling

The functional profiling of the selected strains indicated promising probiotic properties that make the strains an important agent in various industrial applications.

#### 3.7.1. Effect of enzymes on enterocin production

**Figure 7.**
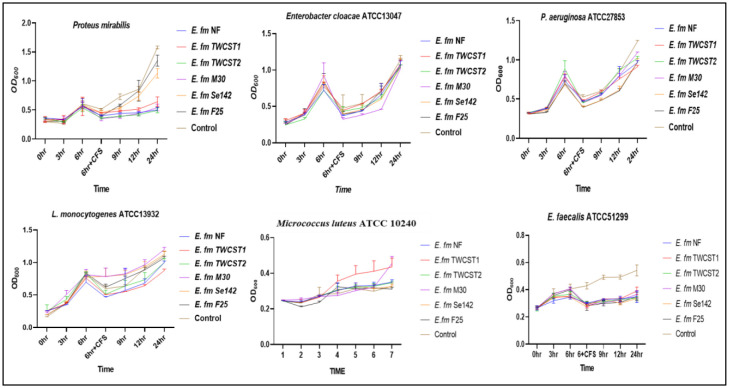
Effect of pepsin treatment on the antimicrobial activity of enterocins produced by the selected *E. faecium* strains.

**Figure 8.**
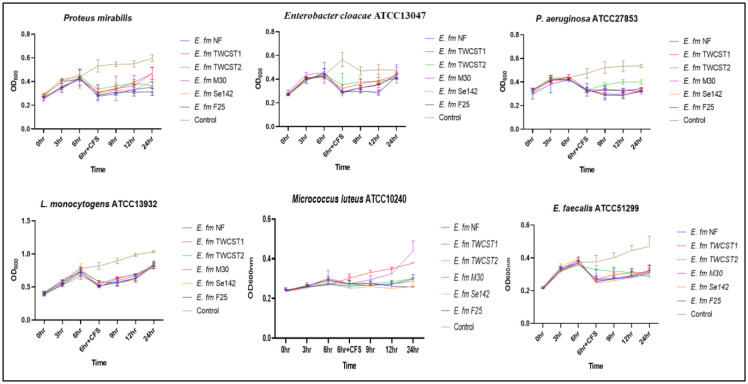
Production of enterocins by the selected *E. faecium* strains and the impact of proteinase K treatment on their antimicrobial activity.

The production of enterocins in the selected strains were identified in another study [12]. The results demonstrated bacteriostatic effects against *Enterobacter cloacae* ATCC 13047, *Pseudomonas aeruginosa* ATCC 27853, and *Listeria monocytogenes* ATCC 13932 when treated with pepsin. In contrast, complete bactericidal activity was observed against *E. faecalis* ATCC 51299, and partial activity was noted against *Proteus mirabilis* (Figure 7). Similarly, treatment with proteinase-K showed bactericidal activity against *P. aeruginosa* ATCC 27853 and *E. faecalis* ATCC 51299, while bacteriostatic effects were observed against *L. monocytogenes* ATCC 13932, *Enterobacter cloacae* ATCC 13047, and *P. mirabilis* (Figure 8).

#### 3.7.2. Screening for arginine hydrolase enzymes

The screening of arginine hydrolase enzymes was evaluated after 48 hrs of incubation. After 24 hrs of incubation, all strains exhibited a color change from purple to yellow, indicating glucose fermentation. Moreover, after 48 hrs of incubation, only *E. faecium* F25 reverted to a purple coloration, indicating positive arginine hydrolase activity, whereas all other strains remained yellow, reflecting the absence of arginine hydrolase production (Figure S5A).

#### 3.7.3. Impact of arginine, glucose, and glycine on growth

**Figure 9.**
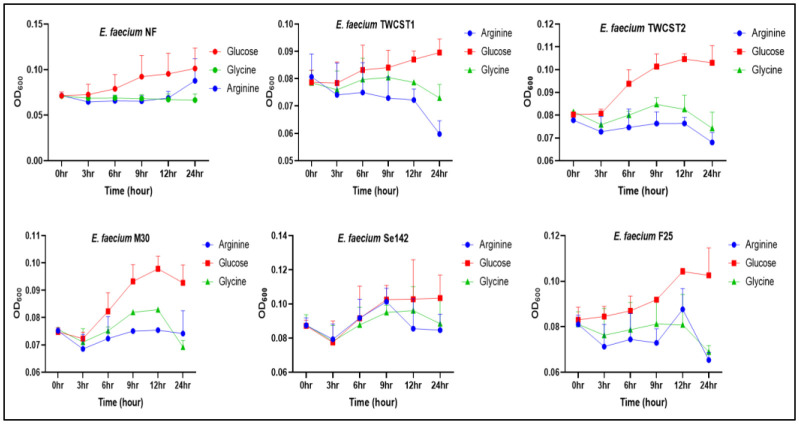
Effect of arginine, glucose, and glycine supplementation on the growth of the selected *E. faecium* strains.

The impact of 25 mM arginine, glucose, and glycine on the selected strains was determined using spectrophotometric measurements and plate assays. The selected concentration was based on other researchers investigating its influence on the arginine deiminase (ADI) pathway and biofilm formation in *Enterococcus faecalis* [42]. Growth curves were generated following overnight incubation, revealing strain-specific responses to the tested compounds. Glucose supported nearly normal growth across all strains, with enhanced growth observed in *E. faecium* TWCST2 and *E*. *faecium* M30. In contrast, arginine exposure led to reduced growth in *E. faecium* TWCST1 and TWCST2, while *E. faecium* F25 and Se142 exhibited irregular growth patterns. Interestingly, *E*. *faecium* NF showed increased growth in the presence of arginine. Glycine supplementation generally resulted in a time-dependent decrease in growth across most strains, except for *E*. *faecium* NF, which maintained stable growth (Figure 9).

The growth performance in terms of cfu/mL was also calculated. In the presence of 25 mM glucose, all strains showed good growth, except *E. faecium* F25, which exhibited lower viability (27 log_10_^7^ cfu/mL). Arginine-supported growth was relatively consistent across strains, with *E*. *faecium* NF demonstrating the highest growth and *E. faecium* F25 the lowest. In contrast, glycine supplementation significantly inhibited the growth of *E. faecium* M30, while having a comparatively mild effect on *E. faecium* F25. A summary of the cfu/mL values is presented in Table 1.

#### 3.7.4. Lactose and glucose consumption (KIA test)

The glucose and lactose consumption profiling were performed using the Kligler Iron Agar (KIA) test. The analysis included evaluation of carbohydrate fermentation and hydrogen sulfide (H_2_S) gas production. After 24 hrs of incubation, the results indicated glucose fermentation, as evidenced by the yellow coloration (acidic pH) in the slants, while no lactose fermentation was observed (Figure S5B). Furthermore, the absence of cracks or gaps in the agar suggested no gas production, and the lack of black precipitate confirmed that none of the strains produced H_2_S gas.

## 4. Discussion

The intrinsic characteristics and unique physiological features of *Enterococcus* species underscore the significance of this genus in both ecological and clinical contexts. With over 80 identified enterococcal species from food, air, water, vegetables, animals, and humans, the genus holds an uplifting position in various industries such as food, dairy, pharmaceuticals, agriculture, and poultry [9]. Despite not being included in QPS and GRAS status microbes, enterococcal species are widely validated and commercialized as probiotics for various applications [15]. Regulatory authorities enabled the functional application of enterococci-based probiotic formulations with adequate safety and precautions. *E. faecium* is one of the leading species of this genus, which has genome plasticity, enterocin production potential, and is generally considered safe. In the previous studies, we explored the probiotic potential of six *E. faecium* strains and their biotechnological properties, isolated locally from various environments of Karachi, Pakistan [1],[12]. Here, we aimed to further explore the probiotic potential of the lead strains and to elucidate their industrial and physiologically relevant properties.

In this study, the previously identified six lead strains were subjected to a series of *in vitro* experiments to explore probiotic and postbiotic potential, free radicals scavenging, and anti-oxidant capacity. During their postbiotic characterization, the slime production potential was elucidated. Slime, an extracellular amorphous substance and regarded as a multifaceted trait that can exert both beneficial and detrimental effects, depending on the microbial strain and environmental context, while generally associated with biofilm formation [45],[46]. Mubarak et al. (2024) documented that more than 80% of enterococcal infections in the U.S. are associated with slime production, highlighting its significance as a virulence factor [47]. Slime can facilitate the bacterial adhesion and show resistance to phagocytosis, which contributes to the persistence of pathogenic strains in clinical environments [46]. In this study, all chosen strains were observed negative for slime production, except *E. faecium* Se142 and *E. faecium* F25. These findings are supporting the previous data, which showed the absence of lipase genes in the selected strains [12]. Additionally, the absence of biofilm formation in the selected strains further confirms the inability of slime production.

Adding to the documented safety assessments, the qualitative biofilm formation was elucidated in this study. Biofilm is considered a virulence trait in enterococcal species, which can enhance the pathogenicity and thus is not considered a wanted property in probiotic strains [1]. In this study, qualitative and quantitative analyses confirmed that the tested strains did not exhibit biofilm-forming capabilities, thereby reinforcing their safety profile for probiotic potential.

Exopolysaccharides (EPS) are high molecular weight biopolymers produced during the metabolic process of microorganisms and classified as homopolysaccharides or heteropolysaccharides [48]. Microbes can produce EPS throughout their growth phases, but maximum production is typically observed at the late logarithmic phase [49]. The EPS production is highly strain dependent and can be influenced by several growth conditions such as growth media, pH, temperature, and carbon/nitrogen ratios. Probiotic bacteria also have the potential to produce EPS, which have differences in composition and charge. Probiotic-derived EPS have various potential beneficial properties, like enhancing gut colonization, protecting against environmental stresses, and exhibiting antioxidant and anti-inflammatory effects [49]. Accordingly, the EPS production observed in the selected strains contributes to their overall functional properties and may significantly enhance their industrial applications.

The Congo red assay is commonly employed to qualitatively detect the EPS and biofilm formation in probiotic strains. Biofilm forming strains typically produce black or dark colonies on Congo red agar plates, whereas non-biofilm producers or weak biofilm producers appear as red or pink colonies [50],[51]. In this study, the appearance of red colonies across all tested strains indicates the absence of biofilm formation, thereby supporting their safety for potential probiotic applications.

Rhodamine dyes, like Rhodamine B, are degraded through various mechanism, including bacterial degradation and photocatalysis. Some probiotic bacteria are shown to have Rhodamine B degradation potential, though it is not a required criteria for probiotics [52]. In the results, all tested strains were negative for degrading Rhodamine B dye.

Phenol red agar is commonly used for microbial carbohydrate fermentation profiling. This assay identifies bacteria based on their ability to ferment specific carbohydrate sources. In the assay, phenol red serves as a pH indicator, changing color from red to yellow in response to acid production resulting from carbohydrate fermentation. This method is particularly useful for characterizing probiotics according to their ability to ferment selected carbohydrates. Fermentative by-products such as lactate, acetate, propionate, and butyrate are key functional traits, as they contribute to microbial metabolic activity and support gut health [24].

Raffinose is a trisaccharide commonly present in plant-based foods like beans, cabbage, and broccoli. It is not digested by humans due to the absence of α-galactosidase enzymes and leading to digestive discomfort including gas production [53]. The ability of *E. faecium* strains to digest raffinose represents a desirable probiotic trait, contributing to improving the digestion and reducing the GIT discomfort. Additionally, raffinose is also considered as prebiotics and thus support the beneficial gut microbes [53]. In this study, all selected strains were observed to metabolize raffinose, supporting their functional probiotic potential and highlighting their applicability in improving the gut health.

Biogenic amines are produced by several probiotic strains depending on the context, while generally their production is considered a virulence trait, particularly when they exceed the threshold level [54]. Biogenic amines are low molecular weight nitrogen-containing compounds, have adverse reactions on the body, and are produced by many LAB species. At lower concentrations BA could influence metabolic and physiological functions and act like neurotransmitters or hormones that mediate immune responses, gut health, and thermoregulation, while at higher concentrations cause adverse events such as headaches, hypotension, hypertension, nausea, palpitations, difficulty in breathing, and sweating [55]. In this study, none of the selected strains demonstrated biogenic amine production, thus strengthening their safety and probiotic applications.

Increased oxidative stress may impairs host redox balance, causing cellular and tissue damage and promoting inflammation [8],[30]. Antioxidants inhibit molecular oxidation and scavenge free radicals, converting them into inactive forms. Inefficient antioxidant mechanisms can exacerbate oxidative stress, contributing to numerous diseases such as diabetes, obesity and cardiovascular illness [56],[57]. Probiotics could scavenge hydroxyl scavenging potential and can produce redox active substances [56]. Microbial cellular component such as exopolysaccharide, bioactive compounds, and antioxidant enzymes are contributing to the bacterial antioxidant potential [29].

The DPPH assay is widely used to evaluate the antioxidant and scavenging potential of probiotic strains [58]. Our findings showed intermediate antioxidant potential using DPPH with a narrow range of variation (20.34 to 27.37%). In other studies, different antioxidant potentials were observed for probiotic strains. For instance, in the study by Kim et al. (2022), the microbial isolated from food showed 2.55–6.88% antioxidant potential against DPPH, Bhagwat & Annapure. (2019) observed in the range of 3.85% to 57.98%, while Pienz et al. (2014) reported higher antioxidant potential (EC_50_ = 3.6) for *E. durans* strain [29],[59],[60]. The lower absorbance indicate the scavenging potential as the DPPH being a stable free radical, can accept the proton of the antioxidant agent [59],[61]. Hence, our findings indicate moderate antioxidant potential and support functional probiotic properties.

Lactic acid bacteria (LAB) species exhibit species-specific free radical scavenging potential, attributed to distinct bacterial components [30]. For instance, Zhang et al. (2022) identified the highest antioxidant potential in the supernatant of *Lactobacillus kefiri* [62], while Lee et al. (2023) identified the highest value in the intact form of *Lactobacillus plantarum* [63]. Similarly, Yang et al. (2023) summarized the high antioxidant potential of cell-free extracts (CFEs) of various LAB strains [64]. In this study, the OH radical scavenging potential of three different cell parts, i.e., intact cells, cell-free supernatants, and cell extracts, was tested and detected in the range of 25% to 39% for the intact form (culture) and from 27% to 34% for the cell-free supernatant. There was very little or no antioxidant potential identified with cell extracts except 1% for *E. faecium* NF and *E. faecium* TWCST2. Cumulatively, these findings indicate that the antioxidant potential is highest in the CFS, followed by the IC, with minimal or no activity in the CFEs. This trend aligns with recent findings by Wu et al. (2025), who reported similar distribution patterns in antioxidant activity among 26 LAB strains isolated from dairy products [30].

The reducing potential in *E. faecium* strains is contributing to their probiotic properties in several ways. Such potential can help to scavenge free radicals and reduce oxidative stress, thus helping maintain overall health [65]. The percent ferric reducing potential of the chosen strain was detected lower in all three components. The highest value was obtained with IC, followed by CFS, while the lowest was observed with cell extracts. In the study of Wu et al. (2025), reducing potential was only observed with ICs, and two strains showed positive results with CFS, while none of the tested 26 strains showed reduced potential with cell extract [30]. Thus, these results have a great coincidence with these findings. Additionally, a notable variation in antioxidant activity among the cellular fractions was observed, with CFSs showing the highest scavenging potential, ICs demonstrating moderate activity, and CFEs showing little or no antioxidant capacity. This differential activity highlights the compartmentalization of antioxidant properties within probiotic strains and highlights the importance of selecting appropriate cellular components for functional applications. Likewise, the study revealed significant ABTS radical scavenging potential of the selected strains, indicating their strong antioxidant capacity. Most of the tested strains exhibited greater than 50% ABTS radical scavenging activity, except for *E. faecium* TWCST2, which showed comparatively lower activity. The observed ABTS scavenging activity supports the probiotic potential of these strains, thus contributing to reducing oxidative stress, maintaining redox balance, and protecting host tissues from free radical–induced damage.

Aggregation and attachment to the mucosal cells are the marked properties of probiotic strains. Visual auto-aggregation, or the visible clumping, that is observed in a culture medium, can be a sign of a probiotic's strength and effectiveness [66]. Probiotic strains with high auto-aggregation abilities often exhibit better adhesion to the gut, helping them to establish in the intestinal environment [32]. Auto-aggregation is considered the ideal property for a probiotic strain, but co-aggregation with pathogenic species is considered lethal [12]. The production of aggregation substances was not detected in these strains, showing no co-aggregation, while visual auto-aggregation was detected with +3 score indexes. Hussain et al. (2023) identified higher-level auto-aggregation potential for these strains, which is supported by this visual determination [12].

Dynamic light scattering (DLS) can be used to obtain information about probiotic cell size distribution, and stability, thus offering a fast and reliable method for characterizing bacterial growth and viability [67]. The change in particle size over time can indicate the cell division and the formation of aggregates of the probiotic bacteria [33]. In this study, DLS was employed to analyze *E. faecium* TWCST1 as a test strain. The analysis revealed an increase in polydispersity index (PDI) from 18% to 31% after 1 hr of incubation, indicating aggregation. A PDI of 31% suggests a relatively narrow size distribution while not highly monodisperse, it indicates reasonable uniformity, which may support the strain's probiotic functionality and reflect its adaptability to the host environment.

Enterococcal aggregation substance (AS) is a surface-bound protein that promotes clumping and aggregation of enterococcal cells. AS production *via* clumping is considered a virulent trait that negatively impacts the strain's probiotic properties [68]. Aggregation substances are involved in biofilm formation and adheres to the surfaces, thus having a role in disease progression [69]. In the underlined study, the tested strains did not exhibit clump formation, suggesting the absence of AS production and indicating enhanced probiotic potential. This observation is supported by the previous PCR analysis, which confirmed the absence of AS-related genes [12].

The fibrinogen-binding potential of probiotic strains is generally considered a positive trait, as it can enhance mucosal adhesion, help in pathogen exclusion, and modulate immune responses. Though, in certain contexts, strong fibrinogen binding may be harmful, as it can contribute to biofilm formation and platelet aggregation [36]. In this study, the tested strains exhibited fibrinogen-binding and non-binding profiles, highlighting their versatility and potential as probiotic agents. This balanced interaction suggests that the strains may be suitable for diverse biotechnological applications while maintaining a good safety profile.

Mucins, a complex glycoprotein, form a mucus layer that lines the gastrointestinal tract and provides a protective barrier. Mucin binding is considered an important probiotic attributes that showed the adhesion potential of a strains to the mucosal cells reflecting the survivability and colonization in the gut conditions [37],[70]. Probiotics can adhere to the mucin layer and colonize in the gut, interact with the immune system, and displace the pathogenic bacteria [71]. The tested strains demonstrated higher cfu/mL counts after 24 hrs of incubation, indicating enhanced mucin-binding capacity and suggesting improved potential for gut colonization.

β-galactosidase is an enzyme that can help in the breakdown of lactose, a sugar present in dairy products [26]. *E. faecium* strains have the potential to produce this important enzyme, contributing to both health-related and industrial applications [72]. The dairy industry is more interested in identifying strains with the ability to produce β-galactosidase as this is related to consumer health, particularly those with lactose intolerance and have the symptoms of hypolactasia [72]. The study demonstrated the moderate production of β-galactosidase enzymes, highlighting their role in food, health, and biotechnological industries. These quantitative data are consistent with the previous qualitative results, which confirmed enzyme production [1].

Tolerance potential is the crucial aspect of probiotic strains, impacting their survival and ability to colonize in the gut. A good probiotic should withstand the harsh conditions of the gastrointestinal tract to reach the intestine and exert its beneficial effects [72]. Acidic/basic and GIT tolerance have already been established in the previous studies [1],[12]. Trypsin are digestive proteases that break down proteins in the digestive systems of animals. A strain's ability to withstand trypsin degradation highlights their survivability long enough to reach the colon and establish its presence. Trypsin tolerance is crucial in exploring the probiotic potential of enterococcal strains because it simulates the harsh conditions of the small intestine, where many probiotics are exposed to digestive enzymes; thus, it is essential for a strain to survive and colonize the gut while tolerating trypsin [73]. In this study, tolerance and percent reduction against trypsin were determined, giving promising results of greater tolerance and very low (strain *E. faecium* M30) or no reduction in the cfu/mL, thus enhancing these strains applications across different sectors.

Alcoholic (ethanol) toxicity majorly occurs due to its interaction with the plasma membrane by interfering with the water-lipid interface, which leads to affecting the cell's integrity, fluidity, and permeability and can also affect the stress response pathways and metabolisms [74]. Ethanol stress is important to explore due to the potential application of the selected strains in industries like beer and wine fermentation. The selected strains showed similar absorbance in the presence of various ethanol concentrations i.e. 5%, 10%, and 15%, although a slight reduction was observed at 15% concentration over time. In the study by Kopit et al. (2014), the tested strains were unable to grow in the presence of 8% ethanol [39]. In this regard, the under studied strains have a greater potential and, hence, more biotechnological applications.

Pancreatic juice tolerance is crucial for exploring the probiotic potential of *Enterococcus* strains because pancreatic enzymes can degrade bacteria in the small intestine. In this study, we explored that all tested strains have greater survivability in the presence of simulated pancreatic juice, although a small change in absorbance was observed after 4 hrs of incubation.

Hyaluronic acid (HA) is a large linear glycosaminoglycan comprised of repeating units of D-glucuronic acid and N-acetyl-D-glucosamine and was observed in the extracellular matrix [40],[75]. HA plays roles in cell migration, adhesion, differentiation, and proliferation, and also has a role in urinary tract, skin and lung infections [40]. Hyaluronic acid interacts with bacteria in several ways. Studies have shown that enterococci based probiotics can be inhibited or killed by HA, while others have shown that they can tolerate or even metabolize HA, reflecting their strain-dependent diversity in metabolism [76]. Tolerance to HA is a positive attribute for *E. faecium* strains, potentially enhancing their probiotic potential. A strain that can tolerate HA exposure might be better equipped to survive and thrive in the gut environment, where HA is present. Thus, in this regard, these strains are showing good probiotic properties.

Enterocins are small proteins and peptides that have antimicrobial activities and have many industrial applications. *Enterococcus* species have the potential to produce one or more enterocin, which make them a useful source of biotechnological and probiotic applications [13]. In this study, the stability and activity of enterocins produced by selected strains, following treatment with pepsin and proteinase K was investigated. The results demonstrated both bacteriostatic and bactericidal effects against various indicator pathogens, highlighting their potential for use in food preservation and therapeutic applications.

Arginine hydrolase activity might contribute to the immunomodulatory effects of some *E. faecium* strains, but its absence is not necessarily detrimental to the overall probiotic potential [77]. The arginine hydrolase production assay was performed to investigate the production of arginine. After 24 hrs of incubation, all tested strains exhibited a yellow color, indicating glucose fermentation. Although, no further color change was observed after 48 hrs in all chosen strains except *E. faecium* F25, suggesting the absence of arginine hydrolase activity in these strains.

Arginine, glucose, and glycine can influence the growth and potentially probiotic properties of a strain. It is documented that arginine can decrease the biofilm formation and enhance the expression of genes related to quorum sensing, metabolism, and polysaccharide production in *E. faecalis* [42]. Growth in the presence of arginine is also suggesting enhance aggregation and cell permeability and promote the lower susceptibility of ceftriaxone and ampicillin [42]. Studies have also demonstrated the important role of L-arginine in metabolism, physiological, and antioxidant defense processes [78],[79]. In this study, the impact of arginine, glucose, and glycine on the growth of the selected strains was elucidated. The results indicated variable growth patterns in all strains, but cumulatively good growth was observed in the presence of 25 mM glucose. The impact of arginine was observed variable as growth was increased with time in *E*. *faecium* NF (a commercial probiotic strain) and *E. faecium* M30. Similar results were found by Snell et al. (2024), who documented that although a relatively high increase in the curve occurred in the presence of arginine, in the end, growth in the presence of glucose is high [42]. Likewise, the impact of arginine *via* plate assay also reflected good growth for *E. faecium* NF, in contrast to the lower growth of *E. faecium* F25. When tested with other substrates, such as glucose and glycine, most strains demonstrated higher growth, except *E. faecium* F25 and *E. faecium* M30 in the presence of glycine. These results suggest strain-specific responses, and notably, the lower growth in the presence of arginine may contribute to the safety profile of the strains, as enhanced growth with arginine has been associated with biofilm formation [80].

The KIA (Kligler Iron Agar) test is used to differentiate bacteria based on their ability to ferment glucose and lactose and produce hydrogen sulfide (H_2_S) and gas, using a medium containing both sugars and a pH indicator. In this study, the tested *E. faecium* strains showed yellow coloration in the slant and butt of the medium, indicating acid production from the fermentation of both glucose and lactose. The acidic slants and acidic butt reflect that lactose-fermenting bacteria could acidify the slants and the tube's depth [43].

## 5. Limitations and future perspective

In this study, we provide a comprehensive analysis of different *in vitro* tests that are helping to elucidate the probiotic potential, biotechnological properties, and industrial application of the selected enterococcal strains. It is important to conduct *in vivo* experiments and genome analysis of lead probiotic strains to fully understand their behavior and applications. Future studies will involve whole-genome sequencing, probiogenomic analysis, and comparative genomic analysis of these lead enterococcal strains, alongside *in vivo* validation using animal models. These efforts will further support the development of commercialized probiotic products.

## 6. Conclusions

The application of probiotics is growing rapidly due to their health promoting effects and industrial applications. *E. faecium* are Gram-positive microbes that include well-characterized probiotic strains with recognized health benefits and diverse industrial applications. We explored the probiotic and biotechnological potential of the selected *E. faecium* strains. In this study, the selected strains were found to be safe, produced beneficial substances including postbiotics, exhibited antioxidant and free radicals scavenging potential, and could ferment raffinose (prebiotic). Additionally, their stress tolerance potential, mucin and fibrinogen binding capacity, and aggregation properties enhanced their survivability and attachments to the mucosal surfaces. These strains were able to grow in the presence of arginine and were slightly affected by glycine supplementation in the growth media. The selected strains exhibited functional traits supporting their potential application in the food, feed, biotechnology, and dairy industries. Altogether, our findings pave the way for the development of novel probiotic formulations.

## Use of AI tools declaration

The authors declare they have not used Artificial Intelligence (AI) tools in the creation of this article.


