# Supplementary material for: Postbiotic production, aggregation properties, binding potential, antioxidants capacity, and functional characterization of the lead Enterococcus faecium probiotic strains
Source: AIMS Microbiol. 2025 Nov 18;11(4):821–54. doi: 10.3934/microbiol.2025035 (PMC12783074; doi:10.3934/microbiol.2025035)
Supplement: Supplementary file 1 [file microbiol-11-04-035-s001.pdf]

AIMS Microbiology

AIMS Microbiology, 11(4): 821–854.
DOI: 10.3934/microbiol.2025035
Received: 15 July 2025
Revised: 14 October 2025
Accepted: 24 October 2025
Published: 18 November 2025

https://www.aimspress.com/journal/microbiology

Research article

Postbiotic production, aggregation properties, binding potential, antioxidants capacity, and functional characterization of the lead *Enterococcus faecium* probiotic strains

Abrar Hussain[1], Muhammad Tanweer Khan[2] and Syed Abid Ali[1,*]

[1] Third World Center for Science and Technology, H.E.J. Research Institute of Chemistry, at International Center for Chemical and Biological Sciences (ICCBS), University of Karachi, Karachi, 75270, Pakistan
[2] Wallenberg Laboratory, Sahlgrenska University Hospital, Bruna Stråket 16, SE-413 45, Gothenburg, Sweden

* **Correspondence:** Email:abid.ali@iccs.edu; Tel: 0092-21-99261720.

Supplement

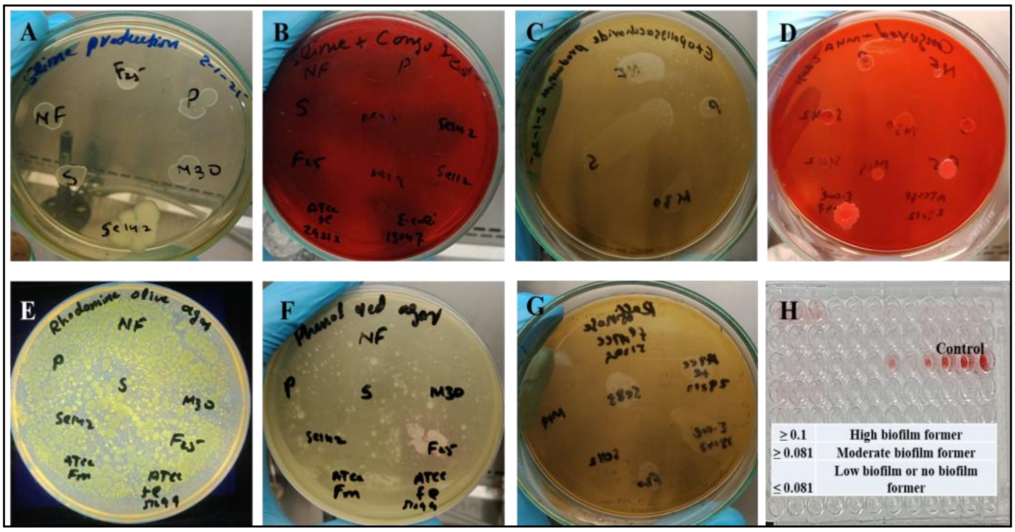

Figure S1. (A) Slime production of the selected *Enterococcus faecium* strains, with *E. faecium* Se142 and *E. faecium* F25 exhibiting positive results (B) Slime production evaluated via the Congo red agar assay (C) Exopolysaccharide (EPS) production, indicated by the appearance of ropy structures (D) Qualitative assessment of biofilm formation using Congo red, where red colony denotes a lack of biofilm formation (E) Rhodamine B dye degradation assay, with the absence of orange coloration indicating negative degradation (F) Phenol red assay, indicating the negative results as no pink color observed (G) Raffinose hydrolysis assay demonstrating positive enzymatic activity of the selected strains (H) Biofilm formation assay on polystyrene microtiter plates, showing no detectable biofilm formation by the tested strain.

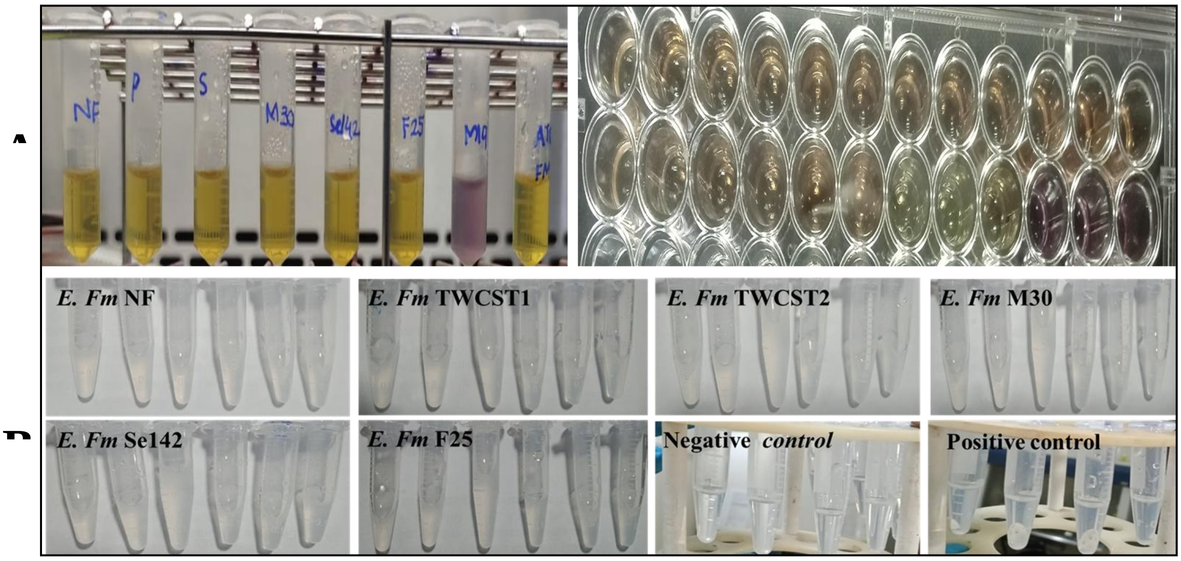

Figure S2. (A) Qualitative detection of biogenic amine production in the selected *Enterococcus faecium* strains (B) Visual assessment of auto-aggregation after 24 hours of incubation at 37 °C; turbidity and visible sedimentation in Eppendorf tubes indicate strong auto-aggregation, with assigned score of +3.

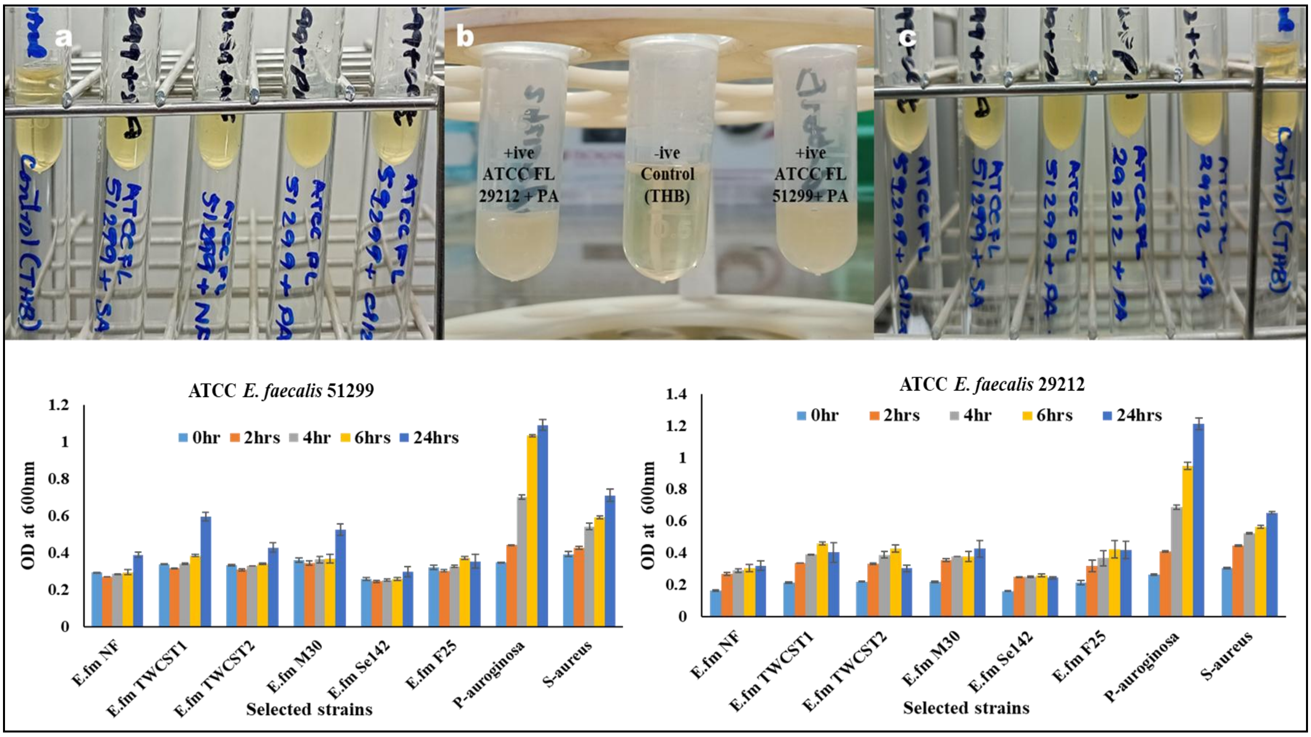

Figure S3. (A) Detection of aggregation substances in the selected *Enterococcus faecium* strains via clumping assay after 24 hours of incubation (a) Visual observation of clumping in selected probiotic strains; (b) Positive and negative controls; (c) Clumping behavior of reference pathogenic strains (B) Quantitative assessment of clumping activity using a spectrophotometric assay against *E. faecalis* ATCC 51299 and *E. faecalis* ATCC 29212.

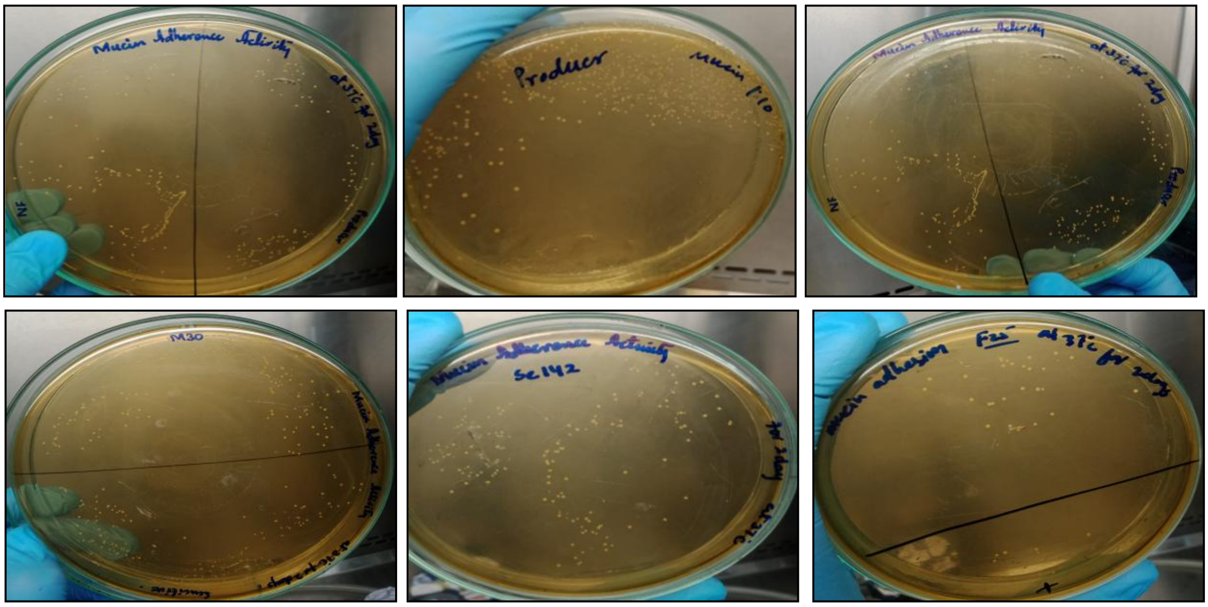

Figure S4. Mucin-binding potential of the selected *Enterococcus faecium* strains. Colony-forming units were counted following 48 hours of incubation at 37 °C to assess adhesion to mucin-coated

surfaces.

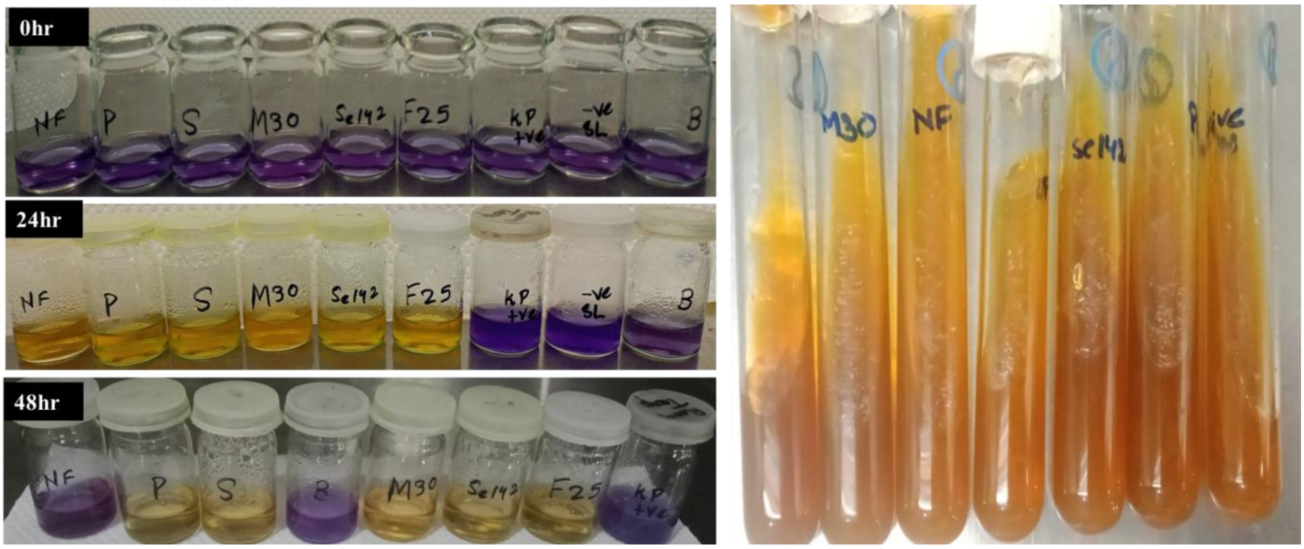

Figure S5. (A) Screening for arginine hydrolase activity in the selected *Enterococcus faecium* strains showed that E. faecium NF is positive while all others are negative (B) Kligler Iron Agar (KIA) test results demonstrating yellow coloration, indicating glucose fermentation by all selected strains.

AIMS Press

© 2025 the Author(s), licensee AIMS Press. This is an open access article distributed under the terms of the Creative Commons Attribution License (https://creativecommons.org/licenses/by/4.0)
